# Glycyrrhizic Acid in the Treatment of Liver Diseases: Literature Review

**DOI:** 10.1155/2014/872139

**Published:** 2014-05-13

**Authors:** Jian-yuan Li, Hong-yan Cao, Ping Liu, Gen-hong Cheng, Ming-yu Sun

**Affiliations:** ^1^Key Laboratory of Liver and Kidney Diseases, Institute of Liver Diseases, Shuguang Hospital Affiliated to Shanghai University of Traditional Chinese Medicine, 528 Zhangheng Road, Pudong New District, Shanghai 201203, China; ^2^Shanghai University of Traditional Chinese Medicine, Shanghai 201203, China; ^3^Department of Microbiology, Immunology & Molecular Genetics, David Geffen School of Medicine, University of California, Los Angeles, CA 90095, USA

## Abstract

Glycyrrhizic acid (GA) is a triterpene glycoside found in the roots of licorice plants (*Glycyrrhiza glabra*). GA is the most important active ingredient in the licorice root, and possesses a wide range of pharmacological and biological activities. GA coupled with glycyrrhetinic acid and 18-beta-glycyrrhetic acid was developed in China or Japan as an anti-inflammatory, antiviral, and antiallergic drug for liver disease. This review summarizes the current biological activities of GA and its medical applications in liver diseases. The pharmacological actions of GA include inhibition of hepatic apoptosis and necrosis; anti-inflammatory and immune regulatory actions; antiviral effects; and antitumor effects. This paper will be a useful reference for physicians and biologists researching GA and will open the door to novel agents in drug discovery and development from Chinese herbs. With additional research, GA may be more widely used in the treatment of liver diseases or other conditions.

## 1. Introduction

The application of natural compounds in the treatment of refractory diseases is a new trend in modern clinical medicine. Because of their satisfactory efficacy in clinic and low toxicity, more natural products are being used as alternative treatments for many diseases. Many hepatoprotective monomers are derived from natural herbs, especially those from China. Glycyrrhizic acid (GA) is an example of one of these hepatoprotective compounds.

The traditional Chinese medicine Gancao (licorice root) is the dried roots of* Glycyrrhiza uralensis* Fisch (licorice),* G. inflata* Bat., or* G. glabra* L. Gancao which was first described in the Chinese book “Shen Nong Ben Cao Jing” in 200 A.D. as an antidote to toxic substances, ache, and other diseases. Gancao can complement other drugs to reduce toxicity and increase efficacy. The traditional use of Gancao involves a decoction of dried plant roots and stems. Some of the possible therapeutic properties of Gancao include antiarthritic [1], antiallergic [2], antiviral [3], antihepatotoxic [4], anticholinergic [5], antiestrogenic [6], anti-inflammatory [6], antileukemogenic [7], and anticarcinogenic effects [8]. It is commonly used for the treatment of acute and chronic liver injury, viral hepatitis, hepatic steatosis, liver fibrosis, hepatoma, viral myocarditis [9], and other diseases like psoriasis [10] or prostate cancer [11].

**Figure 1:**
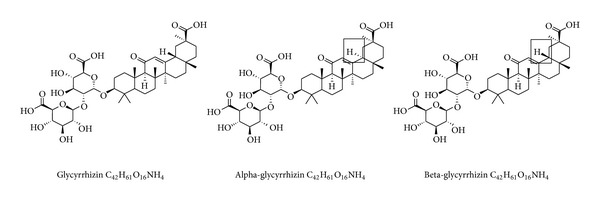
Chemical structure of glycyrrhizin (GA) and its derivatives.

**Figure 2:**
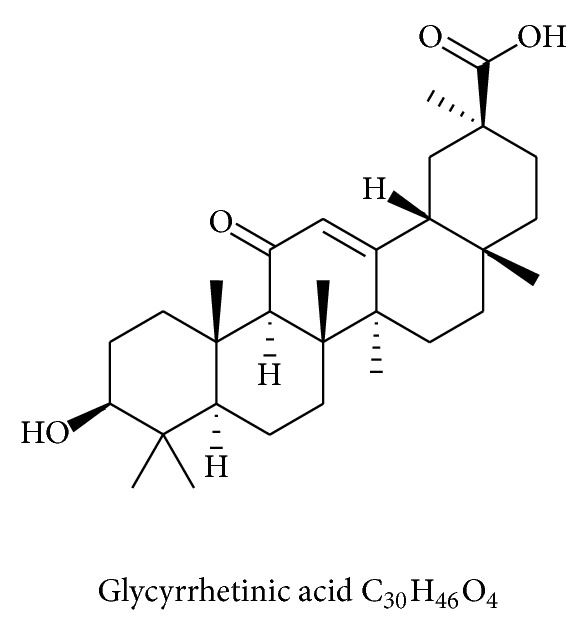
Chemical structure of glycyrrhetinic acid.

The known chemical components of Gancao include saponins (mainly glycyrrhizin (GA), 3.63–13.06%), flavonoids (1.5%), coumarin, alkaloids, polysaccharides, sitosterol, and amino acids [12]. GA (Figure 1) and glycyrrhetinic acid (Figure 2) are well-characterized components of Gancao. GA has been developed as a hepatoprotective drug in China and Japan. GA can generate glycyrrhetinic acid through metabolic processes in the human body. Therefore, the pharmacological effects of GA are essentially the same as glycyrrhetinic acid [13]. GA, also called glycyrrhizin, is a triterpene glycoside from licorice root (*Glycyrrhiza glabra*) and consists of one molecule of 18*β*-glycyrrhetinic acid and two molecules of glucuronic acid (18*β*-glycyrrhetinic acid-3-O-*β*-D-glucuronopyranosyl-(1 → 2)-beta-D-glucuronide) [14, 15]. Glycyrrhizin is considered to be the major active component of Gancao as demonstrated by studies with experimental animal models [16] and clinical studies [17]. GA has been used clinically for more than 20 years in patients with chronic hepatitis in China and Japan [18] and shows a satisfactory therapeutic effect in many other diseases. GA is also widely used as a sweetening and flavoring agent in food.

GA is a main substance of licorice, which is one of the most important substances utilized as traditional medicine for almost 2000 years. Moreover, GA was reported to have antiallergic, antiviral, and anti-inflammatory activities. GA was also found to suppress the rise in fasting blood glucose and insulin levels and improve glucose tolerance. Additionally, GA may act as an antidiabetic substance without inducing side effects, although the mechanism is unclear [19].

**Figure 3:**
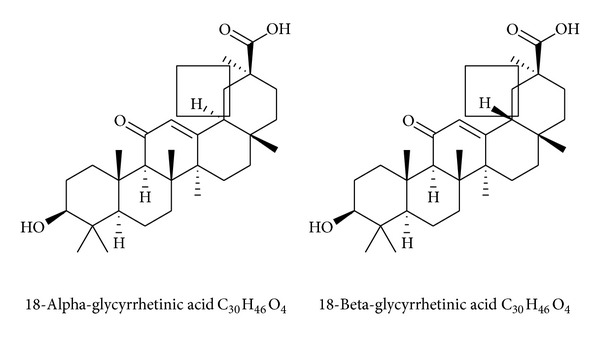
Chemical structure of 18*α*-glycyrrhetinic acid and 18*β*-glycyrrhetinic acid.

GA can form two epimers: *α*-GA and *β*-GA (Figure 3). *α*-GA is derived from *β*-GA by isomerization, and the *α*- and *β*-forms differ only in their C_18_–H–,* trans*-, and* cis*-configuration, respectively. Some scholars examined their distribution characteristics in rat tissue and found that the concentrations of *α*-GA in the liver and duodenum were significantly higher than those of *β*-GA after* i.v.* administration. However, the concentrations of *α*-GA in the other tissues were lower than or similar to those of *β*-GA and declined rapidly. This indicates that the protective and anti-inflammatory effects of *α*-GA on the liver may be better than those of *β*-GA [20].

Several clinical studies reported that GA was efficacious in the treatment of various types of inflammation (mainly in liver [21–30] (Table 1), but also in lung, kidney, intestine, and spinal cord [31]). The most common use of GA is in the treatment of liver disease [32]. GA can reduce steatosis and necrosis of liver cells significantly [33] to inhibit the inter-interstitial inflammation and liver fibrosis and promote cell regeneration. GA has few side effects and is therefore considered to be a drug worth attention and promotion for liver disease.

## 2. Mechanisms of GA Effects

### 2.1. Inhibition of Hepatic Apoptosis and Necrosis

Tumor necrosis factor-alpha (TNF-*α*) is an important cytokine, which is a key mediator of hepatic apoptosis and necrosis in LPS/D-GaAlN-induced liver failure [34]. Plasma TNF-*α* level is also elevated in patients with chronic hepatitis caused by hepatitis B viral [35] and acute alcoholic hepatitis [36]. Therefore, TNF-*α* plays a key role in the pathogenesis of not only endotoxin-induced experimental liver injury but also many human liver diseases. Caspase-3 activation is an indicator of almost all apoptosis systems [37]. GA has anti-inflammatory and antiapoptotic effects via suppression of TNF-*α* and caspase-3 and these are used to explain the hepatoprotective effect of GA (Table 2) [38]. GA also significantly inhibits the release of cytochrome C from mitochondria into the cytoplasm. The anti-inflammatory activity of GA may rely on the inhibition of release of TNF-*α*, myeloperoxidase activity, and translocation of nuclear factor-*κ*B (NF-*κ*B) into the nuclei. GA also upregulated the expression of proliferating cell nuclear antigen, implying that it might be able to promote regeneration of liver injury [39]. Activated Kupffer cells are involved in ischemia-reperfusion- (I/R-) induced liver injury and high-mobility group box 1 (HMGB1) production. GA was shown to inhibit HMGB1 production by Kupffer cells and prevented I/R-induced liver injury [40]. GA could also alleviate I/R-induced [41] and spinal cord [42] injury via this mechanism. In addition, GA conjugates free radicals, which might explain the protective action of GA [43]. For example, GA can be an effective chemopreventive agent against lead acetate mediated hepatic oxidative stress in rats because it binds lead [44]. In concanavalin A- (ConA-) induced mouse model, GA alleviated ConA-induced inflammation and fibrosis progression in livers via inhibition of CD4+ T cell proliferation in response to ConA via the Jun N-terminal kinase (JNK), extracellular signal-regulated kinase (ERK), and phosphoinositide 3-kinase (PI3K)/AKT pathways [45].

### 2.2. Anti-Inflammation and Immunity Regulation

GA suppressed interleukin-6 (IL-6) and TNF-*α* production induced by the lipid A moiety of lipopolysaccharides (LPS) in RAW264.7 cells. It inhibited lipid A-induced NF-*κ*B activation in Ba/F3 cells expressing toll-like receptor 4 (TLR4)/myeloid differentiation protein-2 (MD-2), cluster of differentiation 14 (CD14), and bone marrow-derived macrophages (BMMs). GA also inhibited activation of mitogen-activated protein kinase (MAPKs), including JNK, p38 protein, and ERK in BMMs. In addition, GA inhibited NF-*κ*B activation and IL-6 production induced by paclitaxel, a nonbacterial TLR4 ligand. It attenuated the formation of the LPS-TLR4/MD-2 complexes, resulting in inhibition of homodimerization of TLR4. Therefore, GA modulated the TLR4/MD-2 complex at the receptor level, leading to suppression of LPS-induced activation of signaling cascades and cytokine production. This indicates that GA can attenuate inflammatory responses and modulate innate immune responses [46]. Moreover, GA can prevent the activation of signal transducers and activators of transcription-3 (STAT-3), reduce the upregulation of intercellular cell adhesion molecule (ICAM-1) and P-selectin expression, reduce formation of poly(adenosine diphosphate-ribose) (PAR) and nitrotyrosine, and reduce polymorphonuclear neutrophil (PMN) infiltration. Some observations suggest that broad anti-inflammatory activity of GA is mediated by interaction with the lipid bilayer, thereby attenuating receptor mediated signaling [47]. GA inhibited the lytic pathway of the complement system and may prevent tissue injury caused by the membrane attack complex. Therefore, GA could be a potent agent for suppressing complement-dependent tissue injury in autoimmune and inflammatory diseases [48]. GA can suppress systemic inflammatory response syndrome (SIRS) associated anti-inflammatory response manifestation via inhibition of CC chemokine ligand 2 (CCL2) production by PMN. It may also have the potential to inhibit anti-inflammatory response-associated opportunistic infections in critically ill patients with severe SIRS [49]. There are also other studies that indicated the same anti-inflammatory mechanisms of GA [50].

### 2.3. Antiviral Effects

The antiviral mechanisms of GA mainly include the inhibition of viral replication and immunity regulation. GA affects cellular signaling pathways such as protein kinase C and casein kinase II and transcription factors such as activator protein 1 and NF-*κ*B. Furthermore, nitrous oxide (NO) inhibits replication of several viruses like Japanese encephalitis virus 4 (a member of the Flaviviridae family), which can also be inhibited by GA. The powerful anti-inflammatory capabilities of GA make it effective in the treatment of various types of hepatitis like viral hepatitis and nonalcoholic hepatitis. GA was found to inhibit the replication of the SARS-associated virus [51]. In the treatment of HCV (hepatitis C virus) infection, GA can inhibit HCV full-length viral particles and HCV core gene expression or function in a dose-dependent manner and have a synergistic effect with interferon [52]. GA is also involved in biliary secretion and excretion. GA can increase hepatic glutathione levels by the inhibition of biliary excretion of glutathione partly through the inhibition of MRP2 [53], an efflux transporter located at the canalicular membrane of a hepatocyte. MRP2 translocates glutathione, LTC4, bilirubin, methotrexate (MTX), glucuronide (e.g., estradiol-17-*β*-glucuronide [E_2_17G]), or sulfate conjugates and other organic anions from a hepatocyte into the bile canaliculus [54–58].

GA can activate certain immune functions, such as IFN production, augmentation of NK cell activity, and modulation of the growth response of lymphocytes via augmentation of IL-2 production [70]. GA can enhance immune function in mice [71]. GA treatment could significantly reduce blood immunoglobulin E (IgE), interleukin-4 (IL-4), interleukin-5 (IL-5), interleukin-6 (IL-6), NO, TNF-*α* levels, and nitrous oxide synthase (NOS) activity dose-dependently. GA could also enhance blood immunoglobulin A (IgA), immunoglobulin G (IgG), immunoglobulin M (IgM), interleukin-2 (IL-2), and interleukin-12 (IL-12) levels in AR mice. Gr-1^+^CD11^+^b cells are responsible for numerous pathological processes such as T cell dysfunction after severe trauma or major surgery, leading to increased susceptibility to infection [72]. These cells exercise an inhibitory effect on MBD-1 production of EKs mediated via the suppressor molecules CCL-2 and IL-10. GA acts as a potent inhibitor of these cells and therefore restores MBD-1 levels. This restoration affects T cell dysfunction [73]. In thermally injured mice, GA regulates the burn-associated type 2 T cell responses to recover IL-12 and make it unresponsive, thus restoring the impaired cells [74]. GA acts as a promoter of the late signal transduction of T lymphocytes for IL-2 production. The balance between augmenting and suppressing effects might be dependent on the level of stimulation and stage of the cell. Therefore, this determines quality and quantity of the immunomodulatory action of GA [75]. In blood and nasal mucosa, GA consumption decreases antioxidant enzyme activity, lipid peroxidation, Glutathione levels, and IL-4 levels and enhances IFN-*γ*, thus protecting the nasal mucosa from oxidative injury and improving immunity activity [76].

GA interferes with some viruses, such as H5N1 [77]. The replication and virus-induced proinflammatory gene expression include inhibition of the virus-induced formation of reactive oxygen species and reduced activation of NF-*κ*B, JNK, and p38, which are redox-sensitive signaling events known to be relevant for replication.

### 2.4. Antitumor Effects

CYP enzymes are mainly found in the liver and bowel wall. They are responsible for the bulk of phase I or oxidative metabolism of xenobiotics including dietary toxins, carcinogens, mutagens, and drugs. Administration of GA was able to significantly induce CYP content, which reduces the incidence of cancer [78]. GA can also protect against aflatoxin-induced oxidative stress. The protective effect is likely from its capacity to inhibit the metabolic activation of hepatotoxin, a critical factor in the pathogenesis of chemical-induced carcinogenicity [79]. O-carboxymethyl chitosan nanoparticles (CMCNP) modified by GA with various substitution degrees can efficiently deliver paclitaxel (PTX) to hepatocellular carcinomas (HCC). CMCNP-GA significantly facilitated the increased accumulation of PTX in hepatic tumor tissues and the targeted delivery of PTX to hepatoma carcinoma cells, which resulted in remarkably enhanced* in vitro* cytotoxicity and* in vivo* antitumor efficacy [80]. In a diethylnitrosamine-treated experimental animal study, as a chemopreventive agent of HCC, modulation of cell proliferation and apoptosis by GA may be associated with inhibition of HCC. Therefore, GA treatment may inhibit the occurrence of HCC [81].

### 2.5. Inductive Effect of Liver Enzyme Activity

Some studies showed that GA has an inductive effect on CYP3A activity. Therefore, clinicians should pay attention to other drugs catalyzed by CYP3A, especially those substrates with a narrow therapeutic range such as cyclosporine A, to avoid possible clinically significant interactions with GA [82]. Some studies revealed that the area under concentration-time curve and the mean retention time of methotrexate (MTX) were significantly increased by GA, which increases the adverse reactions of MTX [83]. MTX is an antifolate agent, anticancer agent, and immunosuppressant and is commonly used for anticancer chemotherapy [84], rheumatoid arthritis [85], and severe psoriasis [86]. The adverse reactions of MTX include nausea, vomiting, diarrhea, and hepatotoxicity [87, 88]. A case report showed that combined administration of GA and cilostazol caused pseudoaldosteronism [89]. Therefore, the concurrent use of GA with MTX or cilostazol is not recommended. One report shows a case of hypokalemic rhabdomyolysis secondary to chronic GA intoxication [90]. GA ingestion could therefore potentially aggravate hypokalemia in patients with chronic laxative abuse [91], indicating that the use of GA in hypokalemia should be treated with caution.

## 3. Other Pharmacological Activities

GA is effective in combating hyperglycemia and associated pathological complications such as hyperlipidemia, abnormal histoarchitectures of different organs, and oxidative stress including hemoglobin-induced iron-mediated free radical reactions. The effects of GA on diabetes-associated changes are almost comparable with those of glibenclamide, a standard antihyperglycemic drug, suggesting a possible use of the herbal agent as a drug to prevent complications of diabetes mellitus [92]. Furthermore, GA regulates renal function through the regulation of water channels [93], and GA administration ameliorates the renal concentrating ability and structural lesions in renal tissues in rats with early-phase of ischemia-acute renal failure [94]. As a reduction inhibitor, GA reduces the therapeutic loss of methylprednisolone produced from methylprednisolone 21-sulfate sodium in the large intestine, thus improving the therapeutic property of the prodrug against inflammatory bowel disease [95]. GA also offers protection from the damage induced by UVB radiation in humans. Therefore, it could be considered as a promising agent for addition to topical formulations for the prevention of skin cancer [96]. GA significantly alleviates asthma symptoms [97], inhibits lung inflammation [98], and relieves acute lung injury [35, 99]. It can directly affect cardiac performance and play a role in myocardial and coronary protection in the presence of cardiovascular diseases [100]. GA may prevent brain tissue damage [101], can be a putative therapeutic drug for neurodegenerative diseases associated with cognitive deficits and neuroinflammation such as Alzheimer's disease [102], and could suppress ocular hypertension with potential therapeutic effects in eye disease [103]. GA improves resistance to* C. albicans* infection by inducing CD4+ T cells, which suppress type 2 cytokine production by Th2 cells [104]. GA inhibits activated macrophage (M2M) generation stimulated with neutrophils. The regulation of neutrophil-associated M2M generation by GA may provide a new therapeutic strategy, which could influence the outcome of certain severe infections in hosts with M2M generation [105].

## 4. Drugs That Include GA

Drugs made with GA have been on the market for many years, and most have important therapeutic uses. Magnesium isoglycyrrhizinate injection (TianQing GanMei, Chia Tai Tainqing, JiangSu, China) is one example of a drug with GA. Magnesium isoglycyrrhizinate is an effective and safe treatment for chronic liver diseases [106] and is capable of slowing down the progress of pulmonary fibrosis [107]. Moreover, diammonium glycyrrhizinate enteric-coated capsules (TianQing GanPing, Chia Tai Tainqing, JiangSu, China) and diammonium glycyrrhizinate injection (GanLiXin, Chia Tai Tainqing, JiangSu, China) are used for acute and chronic hepatitis associated with elevated alanine aminotransferase. Stronger neo-minophagen C (SNMC, Minophagen Pharmaceutical, Tokyo, Japan) is often used in the treatment of chronic liver disease and can improve liver dysfunction [60]. SNMC is a compound GA tablet that includes GA (2 mg) with glycine acid (20 mg) and L-cysteine hydrochloride (1 mg). SNMC has anti-inflammatory, antiallergic, steroid-like, anticomplementary, and immunoregulatory effects.

## 5. GA Combined with Matrine

GA combined with matrine (Mat) can improve CCL4-induced liver fibrosis effectively. This is evidenced by lower levels of collagen, hyaluronic acid (HA), and laminin (LN), less hepatic stellate cells (HSC) proliferation, collagen I, and HA levels secreted by HSC* in vitro* with combined therapy compared with GA or Mat alone. GA combination with Mat could protect liver cells and inhibit hepatic fibrosis and may therefore be a safe and effective strategy for improving hepatic fibrosis [108]. In an animal model, GA combined with Mat reduced the mortality of acetaminophen overdosed mice, attenuated acetaminophen-induced hepatotoxicity, and reduced the number and area of y-GT positive foci, thus protecting liver function and preventing HCC from occurring [109]. Additionally, the combination of GA and cyclosporine was an effective treatment for nonsevere aplastic anemia [110].

## 6. Common Derivatives of Glycyrrhizin

Glycyrrhetinic acid (3*β*-hydroxy-11-oxo-oleana-12-en-28-oic acid), the aglycone of GA, stimulates glucose-induced insulin secretion in isolated pancreatic islets. Glycyrrhetinic acid treatment enhances plasma insulin levels and reduces the levels of gluconeogenic enzymes in liver. It is a pentacyclic triterpene acid with numerous biological activities, including anti-inflammatory [63], antiviral [64], antiallergic [65], and antitumor proliferative effects [66].

Glycyrrhetinic acid restrains the proliferation of skin tumors in mice and human breast cancer cells (MCF7) and induces apoptosis of cancer cells. The mechanism of apoptosis might be via increased free Ca^2+^ level in the cells [111]. Mizushina et al. [112] demonstrated that glycyrrhetinic acid potently inhibited the activity of mammalian polymerases, including pol *λ*. Glycyrrhetinic acid also reduced TNF-*α* production and NF-*κ*B activation and suppressed mouse ear inflammation stimulated by tissue plasminogen activator. Therefore, glycyrrhetinic acid could be an anti-inflammatory agent based on pol *λ* inhibition.

Another licorice acid derivative is 18*β*-glycyrrhetic acid. The triterpene structure of the HMGB1-binding compound is capable of binding to HMGB1 and altering its proinflammatory properties, inhibiting HMGB1-dependent cyclooxygenase (COX) 2 induction [113]. 18*β*-glycyrrhetic acid has significant antiviral activity against rotavirus replication* in vitro*, and studies to determine whether 18*β*-glycyrrhetic acid attenuates rotavirus replication* in vivo* are underway, although the exact mechanism is unclear. However, some reports show that 18*β*-glycyrrhetic acid inhibits NF-*κ*B activation, which has been interpreted as 18*β*-glycyrrhetic acid-mediated regulation of the inflammatory response [114]. 18*β*-glycyrrhetinic acid can also inhibit the activity of tyrosine and prevent melanin growth and whitening. Some reports show that 18*β*-glycyrrhetinic acid is likely responsible for amelioration of dysfunction of glutamate transport in astrocytes, and the inhibition of protein kinase C activity might be related to its pharmacological efficacy [67].

## 7. Conclusions and Future Perspectives

This review summarized the efficacy of GA in liver disease from clinical trials and its mechanisms of action* in vitro* and* in vivo*. Studies indicate that GA could modulate various molecular pathways in liver disease. There are numerous patents for drugs including GA (Table 3). Studies described here highlight the use of GA as a novel chemopreventive agent for liver injury. It is expected that future studies with GA will help to define various molecular mechanisms and targets for inflammation and steatosis. At present, the number of multicenter, large sample, randomized, double-blind, controlled chemoprevention clinical trials with GA is very limited. Extensive clinical research is warranted to evaluate the safety and chemopreventive efficacy of GA alone or in combination with chemotherapy agents.

## Figures and Tables

**Table 1:** Clinical trials using compound glycyrrhizic acid injection.

Experimental drugs	Dose and course of treatment	Combined medication	Case/control	Disease type	Indications and symptoms	Efficacy	Positive control	Side effect	Reference
Glycyrrhizin	200 mg + NaCl 100 mL, i.v., q.d., 4 weeks	Prednisolone (20–60 mg)	31/14	Acute onset autoimmune hepatitis (AIH)	Fever, general malaise, fatigue, nausea, vomiting, and right upper quadrant discomfort	Recovery rate was higher in the SNMC group than in the SNMC + CS group (*P* = 0.035)	Glycyrrhizin and corticosteroids (CS)	None	[21]

Glycyrrhizin	200 mg, i.v., q.d., 52 weeks	None	374/129	Chronic hepatitis C	Inflammatory effect	The proportion of patients with ALT reduction ≥50% after 12 weeks was significantly higher with 5×/week glycyrrhizin (28.7%, *P* < 0.0001) and 3×/week glycyrrhizin (29.0%, *P* < 0.0001) compared with placebo (7.0%).	Placebo-controlled	None	[22]

Glycyrrhizin	200 mg + NaCl 100 mL, i.v., 6c/week, 4 weeks	None	69/13	Chronic hepatitis C	HCV and HCV-RNA positive; serum ALT at least 1.5 times; liver fibrosis or cirrhosis	The mean percentage ALT decrease from baseline at the end of treatment was 26% and 47% for the three times per week and six times per week treatment group, respectively (both *P* < 0.001 versus placebo)	Placebo-controlled	None	[23]

Glycyrrhizin	200 mg + NaCl 100 mL, i.v., q.d. for 8 weeks, then 2–7c/week for 2–16 years	None	453/109	Hepatocellular carcinoma (HCC) occurs in patients with hepatitis C virus-RNA positive chronic liver disease	Inflammatory effect after HCC	Patients treated with SNMC; the 10-year HCC appearance rates in histologic Stages I, II, and III were 3%, and 13%, respectively	Other herbal medicines	None	[24]

Glycyrrhizin	200 mg + NaCl 100 mL, i.v., q.d., 0.1–14.5 years	None	1249/796	Interferon-resistant hepatitis C	Chronic hepatitis effect	Crude carcinogenesis rates in the treated and untreated group were 13.3%, 26.0% at the fifth year and 21.5% and 35.5% at the 10th year, respectively (*P* = 0.021)	Others without glycyrrhizin therapy	Hypertension skin rash without itching	[25]

Diammonium glycyrrhizinate	150 mg + 5–10% glucose injection liquid 250 mL, i.v., q.d., 1 month, 1-2 cycles	None	629/127	Chronic hepatitis, liver cirrhosis	Fatigue, gastrointestinal symptoms, and discomfort in liver area	After 17-day therapy, there are 93.3% patients with ALT normal level in treating group, but 73.3% in contrast group (*P* < 0.05). At day 10, the patient'srecovered normal SB were 86.7% in treatment group, but that was 40% in contrast group (*P* < 0.01)	Compound ammonium glycyrrhetate injection	Headache, facial edema, and blood pressure increased	[26]

*β*-glycyrrhetinic acid	80 mg + 10% glucose injection liquid 250 mL, i.v., q.d., 4 weeks, 100 mg, p.o. tid, 12 weeks	None	80/40	Chronic Hepatitis B	Chronic hepatitis effect	Compared with control group, the TBil, ALT, AST, HA, and IVC are significantly ameliorated in treatment group (*P* < 0.01)	*α*-glycyrrhizic acid	Edema, blood pressure increased, and serum potassium mildly low	[27]

Magnesium isoglycyrrhizinate	80 mg + 10% glucose injection liquid 250 mL, i.v., q.d., 4 weeks	Hepatoprotective drugs	80/40	Chronic Hepatitis B	Fatigue, gastrointestinal symptoms, and discomfort in liver area	Compared with control group, the TBil, ALT, AST are significantly ameliorated in treatment group (*P* < 0.05)	Diammonium glycyrrhizinate injection	Headache,and blood pressure increased	[28]

Magnesium isoglycyrrhizinate	150 mg + 5–10% glucose injection liquid 250 mL, i.v., q.d., 4 weeks	None	60/30	Chronic severe hepatitis	Fatigue, gastrointestinal symptoms, discomfort in liver area, and yellow urine	Compared with control group, the TBil, PTA, ALT, and AST are significantly ameliorated in treatment group (*P* < 0.01)	Hepatocyte generation drugs	None	[29]

Magnesium isoglycyrrhizinate	150 mg + 5–10% glucose injection liquid 250 mL, i.v., q.d., 2 weeks	None	56/28	Liver lesion induced by chemotherapy in cancer	Liver injury effect	Compared with control group, the TBil, PTA, ALT, and AST are significantly ameliorated in treatment group (*P* < 0.01)	Diammonium glycyrrhizinate injection	None	[30]

GA: glycyrrhizic acid; TBil: total bilirubin; IVC: type IV collagen; ALT: alanine aminotransferase; AST: aspartate transaminase; PTA: prothrombin time activity.

**Table 2:** Mechanism of action of glycyrrhizin compound chemotherapy.

Compound	Pharmacological activities	Mechanisms of action	Reference
Glycyrrhizic acid	Anti-inflammatory antiviral inhibition of hepatic fibrosis	Regulating the expression of inflammation-related factors; inhibition replication of viral mRNA	[46–49] [51, 59] [45]

Compound glycyrrhizin tablet	Improving the liver dysfunction augmented the entire cytotoxic function mediated by hepatic lymphocytes inhibiting the cascade leading to apoptosis	Regulating the expression of inflammation-related factors; promoting the growth of hepatocyte; inhibition replication of viral mRNA	[60] [61] [62]

Glycyrrhetinic acid	Anti-inflammatory antiviral antiallergic antitumor proliferation	Regulating the expression of inflammation-related factors; inhibition replication of viral; inhibition of the expression of sensitizing factors and tumor-associated factor;	[63] [64] [65] [66]

18*β*-glycyrrhetic acid	Antiviral anti-inflammatory	Regulating the expression of inflammation-related factors; inhibition replication of viral mRNA	[67]

Diammonium glycyrrhizinate	Anti-inflammatory, resistance to biologic oxidation and membranous protection neuroprotective effect	Regulating the expression of inflammation-related factors; regulating the enzymatic reactions' related oxidation	[68]

Dipotassium glycyrrhizinate	Anti-inflammatory	Regulating the expression of inflammation-related factors	[69]

**Table 3:** Patents of glycyrrhizin extracts.

Patent	Patent number
Acetylated 18-alpha glycyrrhizic acid and preparation method thereof	CN102351937 A
Ammoniated glycyrrhizin modified sweetened beverage products	US2008226787(A1)
Application of beta-glycyrrhizic acid and derivatives thereof for radiation protection	CN102206242 A
Application of glycyrrhetinic acid and glycyrrhizic acid in preparing medicaments for preventing or treating pulmonary fibrosis	CN101919870 B
Application of glycyrrhizic acid and glycyrrhetic acid in preparing medicine for inflammatory enteropathy	CN1846705 A
Application of glycyrrhizic acid in preparation of sunitinib malate cardiotoxicity reduction drug	CN103285020 A
Application of glycyrrhizic acid on treating dilated cardiomyopathy cardiac remodeling and cardiac dysfunction	CN102247392 A
Application of glycyrrhizic acid, glycyrrhetinic acid, or salt thereof as well as gel composition and preparation method for gel composition	CN102614213 A
Application of glycyrrhizic acid and its breakdown product glycyrrhetinic acid for the manufacture of a medicament for the treatment of inflammatory bowel disease	US2010087385(A1)
Aqueous pharmaceutical solutions with trisubstituted glycyrrhizic acid salts	EP1226831 A1
*Aspergillus niger* bacterial strain and glycyrrhizic acid used for production thereof	CN101255401 B
Berberine glycyrrhizic acid enantiomer salt and preparation method and usage thereof	CN101747405 A
Biological extraction process of glycyrrhizic acid	CN101067146 B
Carboxymethyl chitosan nanoparticles modified with glycyrrhizic acid, preparation method, and application thereof	CN102357079 A
Chitosan glycyrrhizic acid nanoparticle and its preparing method	CN1586488 A
Composite glycyrrhizic acid amino acid injection and preparation method as well as applications thereof	CN101669962 A
Compositions containing glycyrrhizin	US4678772(A)
Compound for the control of herpes simplex virus using glycyrrhizic acid, lipoic acid, allantoin, and slippery elm	US2011229584(A1)
Compound glycyrrhizin capsule composition	CN103230407 A
Compound glycyrrhizin soluble powder for livestock and preparation method thereof	CN102526082 B
Dispersed compound tablet of glycyrrhizic acid and glycyrrhizinate and its preparing process	CN100386086 C
Enteric-coated formulation of glycyrrhizic acid and its salt and its preparing method	CN1274309 C
Film-coated tablet of glycyrrhizinic acid monopotassium salt and method for preparing the same	CN100341515 C
Glycyrrhizic acid compounds as foamer in chemically derived surfactant-free dentifrice	US2008274062(A1)
Glycyrrhizic acid and its derivative used as RANTES inducer	CN1498623 A
Glycyrrhizic acid antibody and its preparing method and use	CN1293097 C
Glycyrrhizic acid aureola dimer mediated targeted medication body as well as preparation method and purpose of glycyrrhizic acid aureola dimer mediated targeted medication body	CN102716488 A
Glycyrrhizic acid composition	CN101081227 B
Glycyrrhizic acid derivatives having amino acid, its preparation method, and medicinal composition containing them	CN1911954 A
Glycyrrhizic acid double salt and preparation thereof	CN100537593 C
Glycyrrhizic acid matrine salt and glycyrrhizic acid marine salt, its preparing method and use	CN100564391 C
Glycyrrhizic acid organic salt phospholipid ligand and preparation thereof	CN102716463 A
Glycyrrhizic acid removal glycyrrhiza flavonoid and medicament composition thereof	CN101747307 A
Glycyrrhizic acid sustained-release dropping pills and preparation method thereof	CN101269020 A
Glycyrrhizic acid transdermal formulation and preparation technique thereof	CN101433529 A
Glycyrrhizic acid, biogastrone acid or its salt, derivative temperature sensing gel rubber, preparation method, and application thereof	CN101292952 B
Glycyrrhizin high-concentration preparation	US2006160754(A1)
Glycyrrhizin or derivatives thereof for treating or preventing severe acute respiratory syndrome (SARS)	US2007099855(A1)
Glycyrrhizin preparations for transmucosal absorption	US6890547(B1)
Glycyrrhizin-free fractions from licorice root and process for obtaining such fractions	US4163067(A)
Inclusion compound of glycyrrhizic acid or its derivative and alkaloid and its preparation method	CN1301717 C
Magnetic resonance imaging contrast medium with glycyrrhizic acid as carrier	CN101002950 B
Medicine composition of glycyrrhizic acid or its salt and reduced glutathione	CN1985987 B
Medicine composition of glycyrrhizic acid or its salt, ginseng and astragalus root	CN1985873 B
Medicine composition prepared mainly from glycyrrhizic acid or its salt, ginseng and glossy ganoderma	CN1985864 B
Method for determining glycyrrhizic acid content in extract after polysaccharide extraction of glycyrrhiza by virtue of vanillin-sulfuric acid	CN102621089 A
Method for extracting and purifying glycyrrhizic acid by ion-exchange fibers	CN102304165 B
Method for measuring paeoniflorin, hesperidin, and glycyrrhizic acid in stomach-nourishing granules	CN103175915 A
Method for producing glycyrrhizin sodium aliphatate or glycyrrhizin potassium aliphatate	CN101830962 B
Method for producing glycyrrhizic acid through enzymolysis	CN102219824 B
Method for separating and purifying glycyrrhizic acid extracting solution through macroporous resin separation	CN103242393 A
Method of preparing 18 alpha type glycyrrhizic acid and its salt using nonhomogeneous phase reaction	CN100522985 C
Nanocapsule containing glycyrrhizic acid medicine and its preparing method	CN1319537 C
Novel glycyrrhizic acid double salt and preparation and application thereof	CN103242392 A
Pharmaceutical antiviral composition comprising glycyrrhizic acid and at least one protein endowed with antiviral activity	US6329339(B1)
Pharmaceutical antiviral composition, comprising glycyrrhizic acid and at least one protein endowed with antiviral activity	CN1114447 C
Pharmaceutical applications of glycyrrhizic acid or salt and derivative thereof	CN102552280 A
Potassium-magnesium-calcium glycyrrhizin	US4176228(A)
Potentiation of chocolate flavor with ammoniated glycyrrhizin	US3356505(A)
Powder injection of compound glycyrrhizic acid glycosides and preparation method thereof	CN101317852 B
Preparation method for trans-glycyrrhizic acid	CN102584928 A
Preparation method of high-purity glycyrrhizic acid	CN103159809 A
Preparation method of glycyrrhizic acid	CN101759757 A
Process for extracting purified glycyrrhizic acid from licorice residue	CN1450081 A
Process for producing glycyrrhizic acid	CN102617694 A
Products sweetened with alpha-glycosyl glycyrrhizin	US4537763(A)
Separation and purification process of glycyrrhizic acid	CN102453075 A
Separation of glycyrrhizic acid from licorice extract by ultrafiltration	US2011196138(A1)
Separation, purification, and concentration device for glycyrrhizic acid extract	CN202909639 U
Silver glycyrrhizic acid and its producing process and use thereof	CN1063184 C
Slow-released compound preparation of glycyrrhizic acid and glycyrrhizinate and its preparing process	CN1857288 A
Sucrose-ammoniated glycyrrhizin sweetening agent	US3282706(A)
Supercritical CO2 extraction method for extracting glycyrrhizinic acid from licorice	CN1136225 C
Technique for extracting glycyrrhizin using hot reflux method	CN103130863 A
The application of glycyrrhizic acid and its breakdown product glycyrrhetinic acid for the manufacture of a medicament for the treatment of inflammatory bowel disease	WO2007093090 A1
The application of glycyrrhizic acid and its breakdown product glycyrrhetinic acid for the manufacture of a medicament for the treatment of inflammatory bowel disease	EP2067476 A1
Ultrasonic extracting method for changing glycyrrhizic acid leaching phase balance	CN101486750 A
Use of glycyrrhetic acid and/or glycyrrhizin for producing cosmetic preparations for tanning the skin	US2009280074(A1)
Use of glycyrrhetinic acid, glycyrrhizic acid, and related compounds for prevention and/or treatment of pulmonary fibrosis	US2012053141(A1)
Use of glycyrrhizin and its derivatives as MCP-1 production inhibitors	US2004138171(A1)
Use of glycyrrhizin and its derivatives as RANTES inducers	US2004142882(A1)
Use of glycyrrhizin for the treatment of standard therapy-resistant hepatitis C patients	WO2004056374 A1
Use of iso-glycyrrhizic acid and salt thereof in treating allergic rhinitis	CN101396368 B
Use of one or more of glycyrrhizic acids for reducing the irritating action of surfactants in cosmetic compositions	US2011015143(A1)
